# An orientation to wellness for new faculty of medicine members: meeting a need in faculty development

**DOI:** 10.5116/ijme.578a.2064

**Published:** 2016-08-04

**Authors:** Garielle E. Brown, Aleem Bharwani, Kamala D. Patel, Jane B. Lemaire

**Affiliations:** 1 Department of Archeology and Anthropology, University of Calgary, Canada; 2 Office of Faculty Development, Cumming School of Medicine, University of Calgary, Canada; 3 Cumming School of Medicine, University of Calgary, Canada

**Keywords:** Wellness, faculty development, work-life balance, orientation

## Abstract

**Objectives:**

To evaluate the format, content, and effectiveness of a newly developed orientation to wellness workshop, and to explore participants’ overall perceptions.

**Methods:**

This was a mixed methods study. Participants consisted of 47 new faculty of medicine members who attended one of the four workshops held between 2011 and 2013. Questionnaires were used to evaluate workshop characteristics (10 survey items; response scale 1=unacceptable to 7=outstanding), intention to change behavior (yes/no), and retrospective pre/post workshop self-efficacy (4 survey items; response scale 1=no confidence to 6=absolute confidence). Mean scores and standard deviations were calculated for the workshop characteristics. Pre/post workshop self-efficacy scores were compared using a Wilcoxon signed-rank test. Participants’ written qualitative feedback was coded using an inductive strategy to identify themes.

**Results:**

There was strong support for the workshop characteristics with mean scores entirely above 6.00 (N=42). Thirty-one of 34 respondents (91%) expressed intention to change their behavior as a result of participating in the workshop. The post workshop self-efficacy scores (N=38 respondents) increased significantly for all four items (p<0.0001) compared to pre workshop ratings. Participants perceived the key workshop elements as the evidence-based content relevant to academic physicians, incorporation of practical tips and strategies, and an atmosphere conducive to discussion and experience sharing.

**Conclusions:**

Participants welcomed wellness as a focus of faculty development. Enhancing instruction around wellness has the potential to contribute positively to the professional competency and overall functioning of faculty of medicine members.

## Introduction

It is well documented that physicians are at increased risk for a number of negative consequences related to the high stress and demanding nature of their jobs.^1^ They display higher rates of burnout, depression, and suicide than those found in the general population and may experience job dissatisfaction, low quality of life, and relationship failure as a result of their career commitments.^1^ Work-related stress consequences such as burnout may also affect physician performance. Burnout has been associated with reduced workplace productivity and efficiency, ordering of unnecessary tests and procedures, reduced time with patients, and poor physician recruitment and retention.^1^ Faculty of medicine members’ wellness may similarly affect the academic institutions in which they work, including the health and medical education systems.

There are few examples of institutional strategies in development or practice that address faculty members’ wellness through faculty development. There are examples of integrated health and wellness learning at the undergraduate medical education level such as the Vanderbilt School of Medicine “Medical Student Wellness Program” and the Saint Louis University School of Medicine’s pre-clinical curricular change designed to promote medical student wellness.^2^^,^^3^ The latter was associated with significantly decreased depression, anxiety, and stress symptoms in participating students.^3^ At the Boston University School of Medicine a combined yoga and meditation mind-body course was offered to medical students and showed increased self-regulation and self-compassion; and students reported increased community, mindfulness, and confidence in using mind-body skills with patients.^4^ 

While the number and type of wellness strategies in medical schools have increased in recent years, for the academics within faculties of medicine, evidence of opportunities for prescribed wellness training remains relatively sparse. At the Mayo Clinic in Rochester, Minnesota, seventy-four practicing physicians participated in a small-group curriculum intervention aimed at promoting physician well-being, job satisfaction, and professionalism.^5^ The intervention was associated with improvements in empowerment and work engagement, and a sustained decrease in rates of depersonalization, emotional exhaustion, and overall burnout. Strong et al. conducted over one-hundred in-depth interviews with leaders in academic medicine and identified five key themes related to work-life balance: the importance of work-life balance for modern physician-researchers; the effect of gender roles; the role of mentoring; the impact of institutional policies; and the perceptions surrounding work-life balance in the culture of medicine.^6^ Results such as these strengthen the notion that faculty members stand to benefit from wellness interventions and highlight the importance of this topic to academic physicians while also identifying challenges they may face.

Faculty development programs for academic physicians are common at North American universities. A recent review of the literature examined the content of these programs and found that faculty development is most frequently aimed at improving teaching effectiveness, scholarship, career development, and leadership.^7^ None of the articles included in this systematic review reported orientation or mentorship of their faculty members to the topic of wellness. This omission may be one of important consequence. A study that surveyed over two-thousand faculty members from twenty-six representative medical schools in the United-States suggested that almost a quarter of all faculty considered leaving academic medicine, with higher rates of attrition among young and new faculty.^8^ Drivers for leaving included high ethical/moral distress, the need for more institutional support, negative effects from the culture or hidden curriculum of medicine, and a lack of collegial relationships; all aspects that may also affect physician wellness.^1^ Work-related stress places academic physicians at risk for adverse outcomes relating to their personal health, careers, and ability to deliver quality patient care. Accordingly, innovation around what constitutes important topics for new faculty education is needed to address and contend with these challenges.

**Figure 1:**
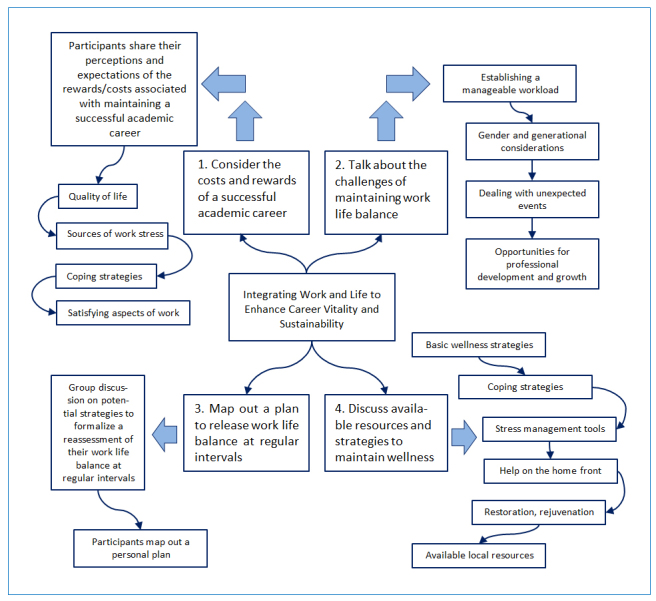
Outline of the orientation to wellness workshop “Integrating Work and Life to Enhance Career Vitality and Sustainability”, developed by the authors

Our institution recently developed a program to welcome and orient new faculty of medicine members and to provide them with practical academic skills. The planning committee considered that instruction around the topic of wellness may be of great value. A wellness workshop was developed and embedded within the program along with more traditional topics such as writing successful grant proposals and scientific writing. Our general goals were to deliver an effective and stimulating workshop on this novel topic, improve wellness literacy for participants, stimulate intention to change behavior for participants, and gather participant feedback in order to better understand how faculty members received and experienced the instruction around wellness. The three-hour workshop, “Integrating Work and Life to Enhance Career Vitality and Sustainability”, (Figure 1), was designed to generate dialogue about the potential rewards and stressors of an academic career and facilitate discussion about strategies to enjoy a productive, balanced, and sustainable work-life experience. Whenever possible, the information presented was based on research findings and peer-reviewed publications from the university’s wellness research team and other related existing literature.

 The purpose of this study was to evaluate the format, content, and effectiveness of this newly developed orientation to wellness workshop, and to explore participants’ overall perceptions.

## Methods

### Research design

We used a mixed methods approach with a quantitative component to evaluate the workshop’s format, content, effectiveness, and a qualitative component to explore participants’ perceptions of the workshop.

### Participants, sampling, and setting

All new physician and scientist hires to the faculty of medicine at our university in western Canada are invited to attend an orientation program that consists of four weekly three hour sessions. We used a volunteer (convenience) sample from those who participated in the orientation to wellness workshop. The data presented reflect the first four sessions (fall 2011, spring and fall 2012, and spring 2013). In total, 81 faculty members registered for the orientation program series, 69 signed up for the wellness workshop, and 47 (68%) attended. The majority held the rank of either Assistant Professor or Clinical Assistant Professor, 17 (36%) were female, and 11 (23%) were non-physician scientists. Considering the small number of participants, further demographic information has been withheld to maintain confidentiality.

### Data collection

We used a written questionnaire to collect quantitative data about the workshop’s format, content, and effectiveness, as well as qualitative data about participants’ perceptions of the workshop. We administered the questionnaire on site at the end of the workshops. Participants’ response rates varied across the study components. Ten survey items (Table 1) measured workshop characteristics (e.g., quality of the facilitator, presentation, and content) on a 7 point Likert-type response scale (range 1=unacceptable to 7=outstanding). Forty-two participants (89%) completed this section. A single yes/no question measured intention to change behavior (i.e., I will change my practice in the future). Thirty-four participants (72%) answered this question. Retrospective pre/post workshop self-efficacy scores of four survey items on a 6 point Likert-type response scale (range 1=no confidence to 6=absolute confidence) rated participants’ confidence in wellness practice abilities (Table 2). Thirty-eight participants (81%) completed this self-assessment. Finally, we asked two open-ended questions to capture what the participants liked most and what they thought should be changed about the wellness workshop.

### Data analysis

We calculated mean scores and standard deviations for the workshop characteristics using Excel. The median pre/post workshop self-efficacy scores were calculated using the Stata 14 statistical package and pre/post scores were compared using a Wilcoxon signed-rank test. Two of the authors independently coded participants’ responses to the open-ended questions using an inductive strategy to identify thematic content. They merged their analyses and resolved differences through discussion.

### Ethical considerations

This research activity is considered exempt from ethics review as per the University of Calgary Conjoint Health Research Ethics Board as it is considered a quality improvement project and a program evaluation initiative. Participants’ attendance to the workshop was voluntary and signified consent to participate in the program. Their responses to the post-workshop survey questionnaires were anonymous. In order to further protect participants’ anonymity, the study results were presented as aggregate data from the first four workshops and the qualitative data were screened for any components that could identify respondents. 

## Results

### Quantitative results

Table 1 shows the mean scores and standard deviations for workshop characteristics (N=42). The mean scores were entirely above 6.00, the lowest being the rating on whether the content was relevant to participants’ practice or not at 6.26 ± 1.06. Participants rated the characteristics of the facilitator highest with enthusiasm rated at 6.64 ± 0.53, interaction with audience at 6.62 ± 0.58, and apparent topic knowledge at 6.62 ± 0.54.

There was expressed intention to change behavior for 31 out of 34 respondents (91%). The qualitative feedback offered further insight where one participant commented “yes! I will try to incorporate many of those suggestions and be more aware of the recent findings,” while another stated they would “pay more attention to wellness.”

**Table 1:** Workshop characteristics mean scores and standard deviations (N=42)

Workshop Characteristics	Mean^*^		SD^†^
The Facilitator			
Enthusiasm		6.64	0.53
Interaction with audience		6.62	0.58
Apparent topic knowledge		6.62	0.54
The Presentation			
Information presented in an organized manner		6.52	0.67
Related the presented information to practical problems		6.36	0.93
quality of support materials		6.40	0.80
The Content			
Volume and complexity appropriate		6.45	0.71
Related content to current evidence in the literature		6.45	0.67
Content was relevant to my practice		6.26	1.06
Overall, how I rate this session		6.52	0.77

*Likert-type scale where 1=Unacceptable to 7=Outstanding; †Standard Deviation

Table 2 shows the results of participants’ (N=38) retrospective self-efficacy ratings in four wellness-related activities prior to and as a result of the workshop. Participants reported significantly higher confidence (p<0.0001) in performing all four of the learning objectives in their post-workshop scores compared to the pre-workshop scores.

**Table 2:** Median retrospective pre and post workshop self-efficacy scores (N=38) for workshop learning objectives and Wilcoxon signed-rank test results comparing pre and post workshop scores

Activity (Learning objectives)	Median pre workshop score	Median post workshop score	Z-score	p-value
Identifying the rewards and costs of a successful academic career	4	5	-5.369	< 0.0001
Meeting challenges of maintaining work-life balance within your career	4	5	-4.700	< 0.0001
Locating available resources and developing strategies that maintain wellness	3	5	-5.467	< 0.0001
Mapping out a plan to reassess work-life balance at regular intervals	3	5	-5.413	< 0.0001

### Qualitative results

Qualitative data were gathered from responses to two open-ended questions on the workshop evaluation questionnaire. Most participants answered using short sentences or point-form comments. The first question asked “What two aspects of this presentation did you like the most?” Seven major themes were identified and included: evidence-based information; practical tips and strategies; the relevant and relatable aspects of the material; the conveyance of content; the relational elements; the workshop atmosphere; and self-reflection and empowerment.

The most frequently cited strength of the workshop was the incorporation of research data as foundations to the key messages, with almost half of participants lauding the use of evidence-based information. Several of them specifically mentioned the inclusion of locally conducted research citing the relevancy not only of the content being evidence-based but also that much of the research came directly out of their own health care environment. One participant noted that they liked, “getting to hear about local studies relevant to the topic” while others described strengths of the workshop as the “evidence-based advice” and “research-based approach.”

Practical tips, strategies, and interventions were perceived as very helpful and were frequently cited as an asset. Participants described how they were provided with “…practical coping skills” and “practical advice on improving balance.”  Similarly, the relevance of the topic and evidence presented as well as the relatability of the presentation were major factors noted as strengths by participants who enjoyed that the workshop had “relevance to real life,” and that the “topic was very relevant to us.” Aspects of the relational elements of the workshop such as sharing stories and experiences with other participants also made the content relatable as participants appreciated hearing “people’s personal experiences/stresses.”

Also highly regarded was the conveyance of the content. This theme includes two sub-themes: the speaker’s skills and enthusiasm and the presentation itself, which included the visuals, slides, anecdotes, and the flow and timing of the workshop. One participant noted “[the presenter] took a topic that could easily have been dry/boring and made it very engaging” while others said “great enthusiasm and interaction by facilitator” and the “presenter incorporated slides and anecdotes to personalize delivery and importance of material disseminated.”

The relational elements of the workshop were frequently cited as being a positive aspect. Two sub-themes emerged from this area: interaction/group discussion and collegial support. One participant said that the “great group discussion” and “supportive colleagues” were the two aspects of the workshop that they enjoyed the most. Other relational elements perceived by the participants included opportunities for “listening to others” and “hearing their colleagues’ … personal experiences [and] stresses.”

The participants described the overall workshop atmosphere in a number of generally positive ways with descriptions such as “refreshing”, “forward thinking”, “unique”, and “outside the box.” Other participants commented on the “open and relaxed atmosphere”, saying the workshop was “candid, frank, and friendly”, and “stress free.” One participant noted that the workshop contained “great ideas for the future of medicine” while another stated that “in no other forum could we address these important issues.” A few participants suggested that the workshop engaged them in self-reflection where one said that the wellness orientation “enlightened me to think of myself.” Others noted that the workshop had given them a “sense of empowerment” or made them “realize how much other physicians feel some things I do.”

The second question asked “What two aspects of this presentation would you suggest be changed in the future?” Most participants left this comment section blank and many explicitly stated “nothing.” Those who did comment most frequently cited the need for the workshop to be more generalizable; they suggested that the research data and intervention techniques presented should apply not only for clinical physician faculty but for the research-based faculty and different career stages as well. “…Including more resident wellness” and “more data for basic scientific lifestyle issues” were two of the suggestions that addressed the issue of specificity. Some participants sought even more practical tips, strategies, and interventions that they could use to address their wellness saying they wanted “more listing of strategies that are working for people,” and “more discussion of interventions… [such as] coping, diet, scheduling, sleep, time management.” Other suggestions from participants were to incorporate a discussion of “more policy,” more hands-on practice with the interventions discussed such as a chance to “learn the 3 minute or 5 minute relaxation strategy,” and one or more follow-up sessions to provide continued support with the faculty over time “to see how we are all coping.”

## Discussion

Our innovative orientation to wellness was deemed a successful pilot intervention given participants’ strong workshop attendance, enthusiastic participation, candid discussions, positive workshop ratings, intention to change behavior, and significantly improved pre/post workshop self-efficacy scores. The participants’ qualitative feedback identified what worked and why, and provided an important source of information for improving subsequent iterations of the workshops.

Evidence-based learning is increasingly being integrated into medical training and physicians respond well to this learning technique.^9^ Updates to workshop content should continue to be evidence-based as our participants strongly endorsed this approach as an effective and necessary tool to teach a novel topic such as physician wellness. The use of local research data was also well-supported and this strategy could be incorporated on a larger scale to improve knowledge uptake for adult learners. The facilitator’s characteristics were rated very highly, but these qualities can be challenging to reproduce in other settings as they are dependent on the facilitator’s skills as a moderator and teacher. This is an important consideration as we train other physicians to deliver the wellness orientation workshop.     

Participants noted that providing concrete examples of practical wellness interventions was a major strength of the workshop. They experienced enhanced literacy about the issue of wellness and learned strategies to help mitigate the potential negative effects of their profession and actively make positive changes for their wellness. Further hands-on sessions, such as demonstrating the practice of stress management techniques, are planned for future workshops. 

The qualitative data suggested that collegial support and camaraderie were important aspects of the workshop. Previous physician wellness research outlines how collegial support helps buffer poor outcomes such as burnout.^10^  This sense of community and a safe workshop atmosphere may have been in part due to the facilitator skills but also due to the smaller group sizes and relational elements of the workshop format, which allowed for plenty of discussion and interaction between participants. Paramount then is ensuring scheduled time to nurture this sense of community through group discussion, integration of personal stories, and informal personal conversations. Participants regarded the workshop as a safe forum for discussion and collegial support, thus we will safeguard small group sizes.

Participants’ responses indicated that they felt empowered, supported, encouraged, and enlightened, and they voiced intention to change behavior to enhance their wellness. Participants’ requests for follow-up sessions speak to the idea of broadening this single wellness orientation into an ongoing wellness mentoring program. Our institution boasts an existing physician wellness initiative that serves to enhance wellness literacy and provide tools to promote and sustain wellness. This initiative targets trainees, academic faculty, and community physicians of all career stages. We plan to enlist this initiative’s resources to provide ongoing group mentorship opportunities. We hope to further assess the impact of this innovation through long-term follow-up with the participants, and by tracking the ripple effect of presenting our experience at medical education and physician wellness conferences.

The mix of both non-clinician researcher and clinician hires in the faculty posed some issues of relevance. Academic clinicians experience different types of work-life stressors than non-clinician faculty members. The workshop’s evidence, strategies and anecdotes, mostly tailored toward the clinicians, was still scored as highly relevant by participants, but the qualitative feedback, although anonymous, suggested that non-clinician academics did not relate as strongly to some of the content. We have now incorporated results of research on the determinants of well-being of non-clinical faculty members in order to address this gap.

The intervention and its assessment as presented here should be interpreted within the context of some limitations. This is a single center mixed methods study. A longitudinal sampling approach to assess the impact of the workshop on participants would offer additional insight. There may have been a response bias among those new faculty members who chose to attend or not attend the workshop, and/or respond or not respond to various components of the survey questionnaire. Future research should focus on the potential for continued mentoring and support options for faculty members at all stages of their careers as well as the possibility of specialty/department specific workshops to improve the relevance and practicality for participants.

## Conclusions

Physician wellness is now seen as a crucial element to the well-functioning of healthcare systems^1^ and presumably to that of faculties of medicine. This paradigm shift is an important one and reflects a more global acknowledgment that wellness is indeed congruent with professionalism. The recent CanMEDS 2015 Physician Competency Framework incorporates a commitment to physician health and well-being into the professional role, linking it to the ability to foster optimal patient care.^11^

Our wellness orientation teaches new faculty members about the importance of wellness behaviors, and equips them with some tools and support systems to promote a healthy, happy, and effective career. Participants welcomed wellness as a focus of faculty development. Enhancing instruction around wellness has the potential to contribute positively to the professional competency and overall functioning of faculty of medicine members. 

### Acknowledgements

The authors would like to thank the W21C Research and Innovation Center, University of Calgary, and the Office of Faculty Development, Faculty of Medicine, University of Calgary for their local support of this project.

### Conflict of Interest

The authors declare that they have no conflict of interest.
